# The milk study protocol: A longitudinal, prospective cohort study of the relationship between human milk metabolic hormone concentration, maternal body composition, and early growth and satiety development in Samoan infants aged 1–4 months

**DOI:** 10.1371/journal.pone.0292997

**Published:** 2024-05-10

**Authors:** Victoria Harries, Jyothi Abraham, Lupesina Vesi, Aniva Reupena, Kima Faaselele-Savusa, Rachel L. Duckham, Richard Bribiescas, Nicola Hawley

**Affiliations:** 1 Department of Anthropology, Yale University, New Haven, Connecticut, United States of America; 2 School of Nursing, National University of Samoa, Apia, Samoa; 3 Obesity, Lifestyle, and Genetic Adaptations Study Group, Apia, Samoa; 4 Institute of Physical Activity and Nutrition, Deakin University, Geelong, Australia; 5 Australian Institute for Musculoskeletal Sciences, The University of Melbourne and Western Health, St Albans, Australia; 6 Department of Chronic Disease Epidemiology, Yale School of Public Health, New Haven, Connecticut, United States of America; Universidade Federal do Rio Grande do Sul Instituto de Ciencias Basicas da Saude, BRAZIL

## Abstract

**Background:**

Current research suggests that energy transfer through human milk influences infant nutritional development and initiates metabolic programming, influencing eating patterns into adulthood. To date, this research has predominantly been conducted among women in high income settings and/or among undernourished women. We will investigate the relationship between maternal body composition, metabolic hormones in human milk, and infant satiety to explore mechanisms of developmental satiety programming and implications for early infant growth and body composition in Samoans; a population at high risk and prevalence for overweight and obesity. Our aims are (1) to examine how maternal body composition influences metabolic hormone transfer from mother to infant through human milk, and (2) to examine the influences of maternal metabolic hormone transfer and infant feeding patterns on early infant growth and satiety.

**Methods:**

We will examine temporal changes in hormone transfers to infants through human milk in a prospective longitudinal cohort of *n* = 80 Samoan mother-infant dyads. Data will be collected at three time points (1, 3, & 4 months postpartum). At each study visit we will collect human milk and fingerpick blood samples from breastfeeding mother-infant dyads to measure the hormones leptin, ghrelin, and adiponectin. Additionally, we will obtain body composition measurements from the dyad, observe breastfeeding behavior, conduct semi-structured interviews, and use questionnaires to document infant hunger and feeding cues and satiety responsiveness. Descriptive statistics, univariate and multivariate analyses will be conducted to address each aim.

**Discussion:**

This research is designed to advance our understanding of variation in the developmental programming of satiety and implications for early infant growth and body composition. The use of a prospective longitudinal cohort alongside data collection that utilizes a mixed methods approach will allow us to capture a more accurate representation on both biological and cultural variables at play in a population at high risk of overweight and obesity.

## Introduction

Leptin, ghrelin, and adiponectin are metabolic hormones that contribute to the regulation of satiety and energy homeostasis. These hormones, also known as adipokines or ‘appetite/satiety hormones’, are influenced by the levels of adiposity and are actively transferred from mother to infant through human milk [1–6]. All three hormones are vital for regulating physiological functions that are sensitive to body composition and developmental programming of hormonal equilibrium throughout life, along with a host of other influences on health. **Leptin** can reduce appetite and increase energy expenditure, while promoting the formation of neural circuits that influence food intake and adiposity in infants [7]. **Ghrelin** stimulates appetite, thereby increasing food intake, and exerts adipogenic activity which contributes to long term regulation of body weight [4, 5]. As leptin can decrease appetite as a signal for satiety and ghrelin increases appetite as a signal for hunger, a balance of both may be optimal to establish homeostasis and regulate sufficient satiety responses [8, 9]. **Adiponectin** regulates insulin sensitivity, glucose and fatty acid metabolism, and has anti-inflammatory effects [4, 6, 7]. Adiponectin is the most abundant metabolic hormone present in human milk and has been found to influence growth and development in infants [2, 4].

From an evolutionary perspective, as the first nutrition that an infant typically receives, human milk serves as a key contribution in maternal signalling to potentially prime the offspring for the environment in which they will develop [10]. Current research supports the theory that maternal/infant energy transfer through human milk influences early nutritional development of the infant and begins metabolic programming that will influence early growth trajectories and eating patterns into adulthood [2, 5]. As a result, breastfeeding has been hypothesized to be protective against obesity later in life. Researching variation in signalling through human milk, predicted to be influenced by maternal body composition, can shed light on the importance of this critical window of early nutrition for setting an optimal phenotype for an infant’s future environment.

However, most research regarding the programming effects of maternal metabolic hormones through human milk have predominantly been conducted among women with healthy weight in high income settings, namely the United States (US), United Kingdom (UK), and Western Europe, or among undernourished women in low-income nations [11]. Research has yet to explore whether the process of lactational metabolic programming happens similarly among women who are over-nourished—reflected in overweight or obesity—and/or have body composition stores that potentially buffer milk composition against external influences. Samoa presents an appropriate population in which to explore this question. Alongside their exceptionally high breastfeeding rates– 94% initiation with 55% continuation to 5-months exclusively [12]–Samoan communities exhibit high levels of obesity with 43.8% of adult males and 58.4% of females having obesity based on international body mass index (BMI) thresholds [13]. Obesity becomes problematic at a young age in Samoa, with approximately 16% of 24-59-month-olds categorized as having overweight/obesity based on global growth standards [14].

Additionally, little research has explored whether cultural feeding practices are an important determinant of infant satiety than milk composition. For example, Samoan mothers believe male infants are hungrier and in need of increased feeding, resulting in later average weaning age compared to females [15]. This sex difference has been attributed to a mix of cultural and biological mechanisms—with cultural expectations of feeding behavior, maternally perceived infant hunger and satiety responses, and metabolic hormone concentrations in human milk differing depending on the infant’s sex [16].

Obesity has a complex etiology and Samoa has a unique evolutionary history in which to investigate it. Ongoing research into the etiology of excess adiposity among Samoans has focused on risk factors such as genetics, diet, and physical activity and has contributed greatly to our current understanding [16–22]. This study will investigate how maternal body composition influences infant circulating hormonal concentration through human milk, and the influence of maternal-infant dyad feeding behaviors and infant satiety on early infant growth and adiposity.

## Materials and methods

### Aims and study design

This study will address two aims: 1) to examine how maternal body composition influences metabolic hormone transfer from mother to infant through human milk, and 2) to examine the influences of maternal metabolic hormone transfer and infant feeding patterns on early infant growth and satiety. These research aims will be accomplished through examining temporal changes in hormone transfers (leptin, ghrelin, and adiponectin) to infants through breastfeeding and the impact on the infant’s hormone concentrations in a prospective longitudinal cohort of *n* = 80 Samoan mother-infant dyads. Participants will be assessed at three time points (1, 3, & 4 months postpartum; ±5 days) to capture periods of key growth and developmental changes. The research will utilize a mixed-methods approach to data collection to capture a more accurate representation of both biological and cultural variables at play in the population.

Pilot research was conducted November 2020 –April 2021, collecting human milk samples and anthropometric measurements from mothers and their infants aged 2–4 months utilizing a cross-sectional study design. Anthropometric measurements included maternal height and weight, and infant length, weight, skinfolds (triceps, subscapular, iliac crest, and thigh), and head circumference. Additionally, the mother received a dual energy x-ray absorptiometry (DXA) scan. The pilot study allowed for training in collecting human milk, body composition measurement, sample transportation, and metabolic hormone sample preparation and analysis techniques to be undertaken in preparation for this research. The pilot research has informed many decisions during the conceptualization of this current study design that will be discussed in later points.

### Ethical approval

Ethical approval was received from Yale University Institutional Research Board (IRB # IRB00011725; Human Research Protection Program) in September 2021. IRB Protocol ID: 2000029993. Local ethics and research approval was received from the Health Research Committee (HRC) of the Samoa Ministry of Health in November 2021.

### Study setting

The study will be conducted in Samoa, an independent Pacific Island nation with a population of over 200,000, of whom ~93% self-identify as having Samoan ethnicity [23]. Samoa consists of two main islands, Savai’i and the smaller—but more populated—Upolu, as well as several smaller islands. The study will be conducted at the Obesity, Lifestyle, and Genetic Adaptations (OLaGA) research center at the Samoa Ministry of Health (MOH), which is situated in the capital city of Apia, on the island Upolu.

### Study population/participants

The study will recruit a sample of *n* = 80 Samoan pregnant and early-postpartum (≤3 weeks postpartum) women residing in Upolu, Samoa, who plan to exclusively breastfeed for a minimum of 4 months. We are aware that mothers may not maintain exclusive breastfeeding, therefore, mothers will remain eligible to continue in the study after enrollment as long as they are giving *any* human milk to their infant at the time of data collection visits. Questions are included in the questionnaire to record information on current feeding practices (exclusive or partial breastfeeding) and these data will be controlled for in later analyses. Samoa has a high prevalence of initiation and duration of breastfeeding [24], however, if a participant is no longer giving any human milk during the 4-month study period they will not be eligible to continue data collection.

Women with a diagnosis of diabetes (type 1 or type 2; ~20% of the population [25, 26]) or a history of gestational diabetes will be excluded from participating, due to the known deregulatory effects of diabetes and antidiabetic medications on serum metabolic hormone concentrations [27, 28]. Additionally, screening using HbA1c (Glycosylated Hemoglobin A1c) will be used to identify women who meet this exclusion criterion but who have not yet been diagnosed. If HbA1c –using point of care A1cNow+^™^ kits (PTS Diagnostics 3021)–is ≥ 6.5%, participants will be ineligible to participate and will be referred for further evaluation by a clinician.

During recruitment we will aim to have an approximately even division of participants by weight status: 50% women with BMI measurements in the healthy/overweight range (18<32 kg/m^2^) and 50% with obesity (BMI > 32 kg/m^2^). The BMI thresholds used are Pacific Islander specific, as opposed to the World Health Organization criteria, to be more sensitive to the body composition of this population [29, 30]. Due to the high prevalence of overweight and obesity in Samoan women [12], equal recruitment across the three weight categories (normal, overweight, and obese) was not met for the normal weight category during the pilot study. As a result, the decision was made to include normal and overweight BMI status in one category for this study. We will aim for an even distribution of infant biological sex to later control for confounding.

Participants will be excluded from participating if they have had a chest x-ray, a computed topography scan, or a dual-energy X-ray absorptiometry (DXA) scan in the 6 months prior to the beginning of the study. This is to reduce unnecessary x-ray exposure as the study will require two DXA scans within the 3-month study period. See Table 1 for further detail on inclusion and exclusion criteria.

**Table 1:** Inclusion and exclusion criteria.

INCLUSION	EXCLUSION
Postpartum Female ≤3 weeks Aged 18–45 years Singleton birth Planning to engage in exclusive breastfeeding for 4 months Non-smoking during pregnancy Samoan ethnicity Fluent Samoan or English language ability Willingness and ability to complete all elements of the data collection	Currently pregnant Diagnosis of Type 1 or Type 2 diabetes History of gestational diabetes Known history of thyroid disorder HbA1c ≥ 6.5% Intent to move away from Samoa during study period A chest X-ray or computed tomography scan in the past 6 months A DXA scan in the past 6 months (prior to beginning this study) Metal or other implants in the body Too many participants in the BMI category** Too many infants of the same biological sex**

Note:

** As we are aiming for an even distribution of maternal BMI and infant biological sex in our sample for purposes of statistical power or potential confounding, these will be monitored throughout recruitment.

### Recruitment strategy

Participants will be recruited via social media (www.facebook.com/YaleOlaga) and posters placed in the local hospitals and well-child clinics within Samoa. Social media is broadly used in the Samoan community and this method has been successfully utilized by the OlaGA research team for past projects. Advertising will ask mothers to contact the research team via the Facebook page or by cell phone to express interest, including a contact number to allow for pre-screening calls to ensure eligibility and arrange a home visit to conduct the informed consent process.

#### Summary of study design and timeline

**Fig 1:**
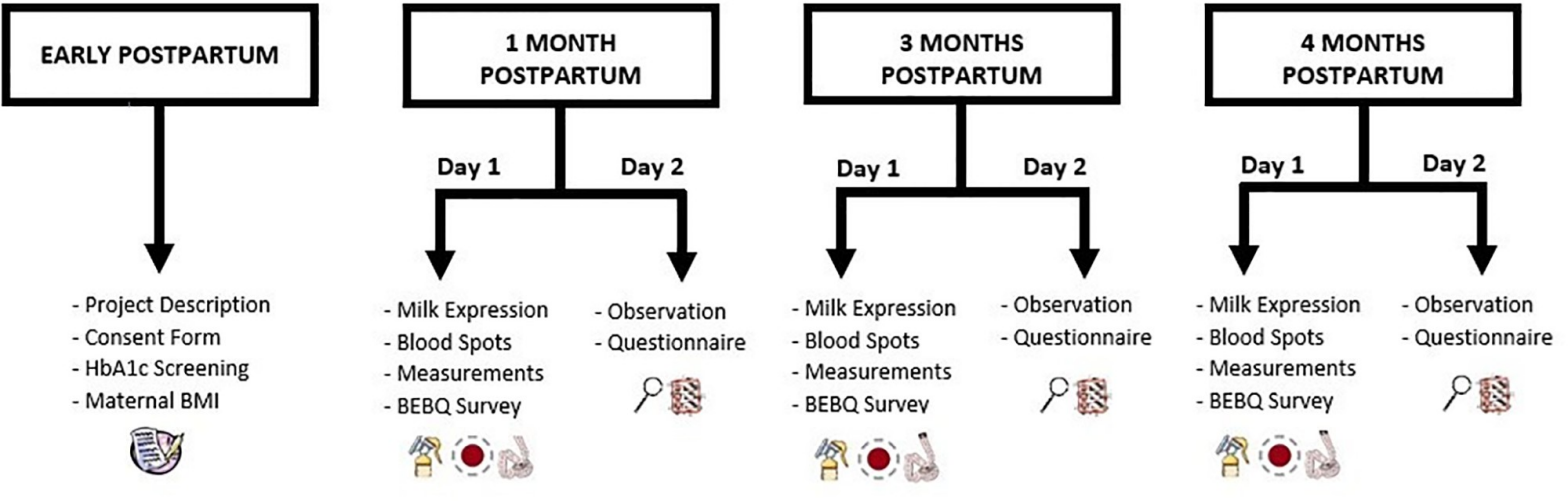
Study design flowchart.

Participation in the study will involve the following steps (in order): 1) a pre-screening phone call during late pregnancy or early postpartum period, 2) a home visit for consent and additional eligibility screening, 3) a research center visit followed by a home visit at 1 month postpartum, 4) a research center visit followed by a home visit at 3 months postpartum, and 5) a research center visit followed by a home visit at 4 months postpartum (Fig 1). The research center visits will take place between 8-10am to control for circadian rhythms of metabolic hormones and the home visits will take place 1–5 days after the research center component (depending on the mother’s availability and preference).

A combination of research center and home visits will be used to ensure that breastfeeding interactions are captured in the dyad’s usual feeding environment and represent typical feeds.

#### Early postpartum visit variables and procedures

*HbA1c screening*. After explanation of the study and signing the consent form, a fingerstick blood sample will be taken from the mother to be analyzed using a point of care A1cNow+^™^ kit (PTS Diagnostics 3021). In the event of a participant recording HbA1c >6.5% (indicative of overt diabetes), they will be informed, referred to clinicians at their nearest community health center, and will no longer be eligible to participate in the study, due to the known dysregulation of human milk composition resulting from maternal diabetes [27, 28]. If the participant does not currently have a physician, the study team will find and refer them to a nearby physician.

*Anthropometric measures*. We will collect weight and height of the mother to calculate maternal BMI (kg/m^2^). This will be used to ensure an equal distribution of BMIs across the sample of mothers. If the requirements for a BMI category are met (~40 participants with normal/overweight or obesity) then new participants with measurements in this BMI category will not be eligible to participate.

*Demographic survey*. We will collect maternal demographic information through a questionnaire. We will ask about age, marital status, educational status, employment, average household income, and parity. These data will be used to control for confounding during data analysis. See Table 2 for more information on questionnaire and survey contents.

**Table 2:** Breakdown of questionnaire and survey contents.

Questionnaire	Number of Items	Visit	Description	References
**Demographic Information**	22	Consent	Demographic information including age, marital status, education, and family income	
**Baby Eating Behavior Questionnaire (BEBQ)**	18	Research Center	Maternal perception of infant appetite, hunger, and satiety during breastfeeding.	Llewellyn et al. (2011) [42], Oyama et al. (2021) [39]
**Infant Feeding and Satiety**	17	Home	Breastfeeding patterns (bouts per day, duration, night time feeding patterns, delivery method), foods/liquids other than breastmilk, contributions by others to infant feeding	Fein et al. (2008) [40]
**Infant Sleep**	2	Home	Location of infant during sleep and who sleeps with the infant on a typical night	
**Maternal Informational Sources**	12	Home	Semi-structured interview. Recognizing signs and signals of infant hunger and satiety, sources of information from which mother’s learnt signals or signs of infant hunger and satiety, use of classes and interest in further information	
**Infant Body Size**	4	Home	Maternal perception of her infant’s body size and ideal body size	Hawley & Gorrepati (2017) [41]

#### Research center visit variables and procedures (Day 1)

*Biospecimen collection*. Human milk and blood spot samples will be collected between 8am-10am during research center visits. Restricting the visit to the morning will control for diurnal variations in the milk hormone levels. Our previous pilot study found that women in this population have a tendency towards a breast that produces more milk, this we will refer to as the dominant breast. Participants will have been asked to fast for a minimum of 2 hours before their laboratory visit and asked not to breastfeed from their dominant breast for the same amount of time. On arrival at the research center, participants will be asked for the time of their last meal, the time they woke that morning, and their infant’s last feed. These will be noted for use as potential confounding factors in later analysis.

Human milk samples will be collected using an electric breast pump in a closed, private area of the research center, with the help of one research assistant trained in lactation consulting with previous experience collecting human milk samples. Only the research assistant and the mother will be present to ensure privacy. Wearing sterile latex gloves, the research assistant will swab the dominant breast with an iodine swab before collection. Next, the milk will be collected into a leak-proof container with a sterile electric breast pump. The research assistant will show the mother how to use the breast pump, if she is not familiar. If the participant expresses discomfort, the position of the breast pump will be adjusted; however, if the participant continues to express discomfort, we will stop milk collection. To prepare the human milk samples for storage, we will invert the container to ensure an equal mixture of fore-, mid-, and hind-milk throughout the sample. The human milk samples will be aliquoted into 5 mL cryovials, labelled with their identifying code, and stored in the OLaGA research center freezer at -80°C for later analysis of hormone levels (leptin, ghrelin, and adiponectin).

The bloodspot collection will be taken from the dyad to measure circulating hormone levels (leptin and adiponectin only; the instability of ghrelin results in the inability to measure through bloodspot collection). Bloodspots will be taken using a finger-prick blood extraction from the mother and a heel-prick blood extraction from the infant onto a filter paper collection card. Sterile latex gloves will be worn by all research team members involved in collection of bloodspot samples and alcohol wipes will be used to clean the areas prior to blood collection. The bloodspots will be dried, placed in a resealable plastic bag containing desiccants, and stored at -80°C.

*Body composition assessments*. Dual energy x-ray absorptiometry (DXA; Lunar iDXA, Encore version 17, General Electric (GE) Healthcare Medicine) will be utilized to assess maternal and infant total body composition at 1 month and 4 months. The body composition outcomes of interest will be fat mass (kg), lean mass (kg), and bone mineral content (g), estimated using the CoreScan application, GE Healthcare Medicine. Participants reporting a CT scan or high dose x-ray or DXA scan within the past 6 months during the eligibility pre-screening will be excluded from the body composition assessment to help reduce the risk of non-clinical expose to radiation. Mothers will be required to take a pregnancy test prior to participating in a DXA scan and will not be eligible to complete the scan (or to continue participation in the study) if a positive test is noted. For the DXA scan, mothers will be asked to change into standard clothing (for example, shorts and t-shirt) with no metal components. A total body scan will be conducted to measure fat mass, lean mass, and bone mineral content in both mothers and infant. Mothers will lay supine with arms and legs straight. To allow for measures of regions of interest spacing blocks will be placed between the mother’s body and arms. Straps will be placed around the mothers’ knees and ankles to allow them to remain still for the entire scan taking approximately 10 mins. In cases where the mother’s body size does not fit within the scan area, the participant will be positioned so that the one half of their body is scanned in a first scan and then the other half is scanned in a second scan. These scans will then be analyzed using a mirror mode, in which the total body composition will be estimated from one half of the body. This technique of analysis has been effective among obese adults in other settings [31] and has been utilized by the OLaGA study team in previous research [32].

For infants a total body DXA scan conducted in the pediatric model will be performed with the infants stripped to their diapers and swaddled in a blanket to help to reduce movement, following the protocol that has been used in previous infant body composition studies conducted by our research team [33, 34]. For measurement of infant bone mineral content, scans will be analyzed less the head, due to the disproportionately large size of an infant’s head in comparison to the rest of their bodies. Infant scans will take approximately 2–3 minutes. Research assistants at the OLaGA research center trained in use of the DXA scanner will oversee all scans.

Additionally, we will collect maternal body fat percentage using Bioelectrical Impedance Analysis (BIA) measures of resistance and reactance with an RJL BIA-101Q device (RJL Systems). BIA measurements will be used, alongside the anthropometric measurements detailed below, to calculate body fat percentage during the Month 3 visit in which a DXA scan is not taken [35].

*Anthropometric measurements*. We will measure maternal weight (kg) and height (cm), using a digital weighing scale (Tanita HD 351; Tanita Corporation of America) and a portable stadiometer (SECA 213, Seca GmbH & Co). Weight and height will be measured in duplicate to the nearest 0.1kg and 0.1 cm, respectively, and averaged. The maternal measurements will be used to calculate maternal BMI (kg/m^2^).

**Fig 2:**
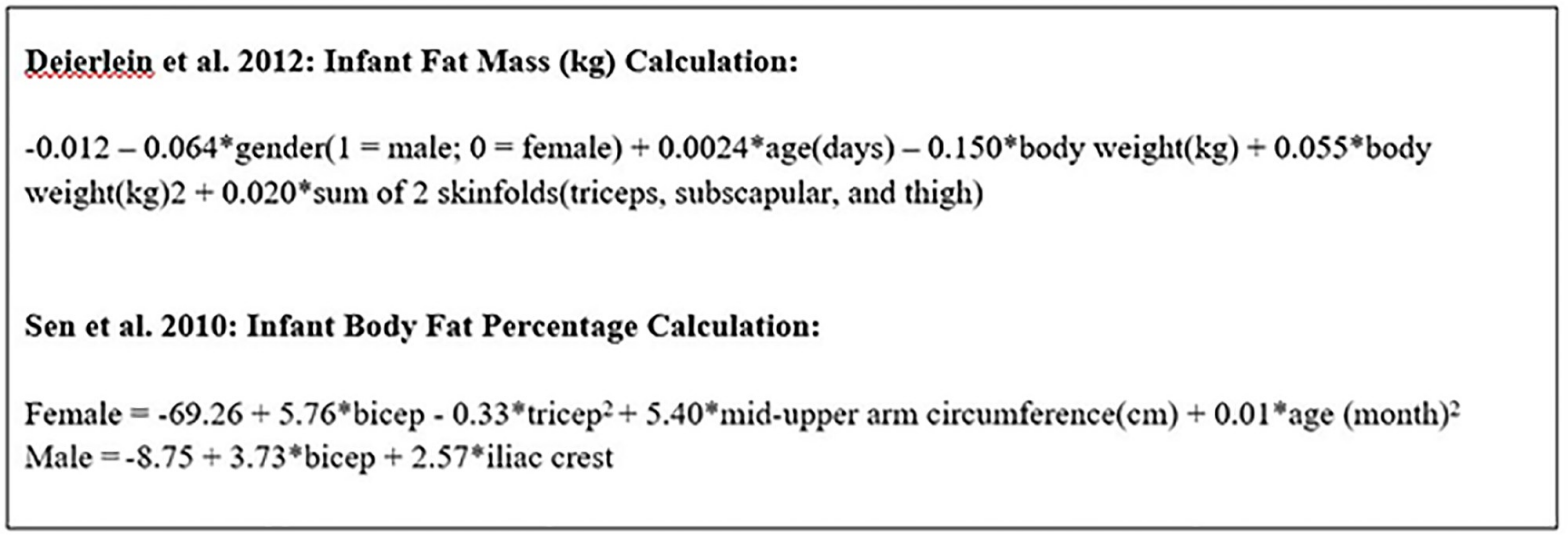
Anthropometric equations for estimating infant fat mass.

Anthropometric measurement of the infant will include weight (g), length (cm), head, mid-upper arm, and abdominal circumference (mm), and skinfold thicknesses (mm). Skinfold measurements includes: triceps, biceps, subscapular, iliac crest, and thigh using calipers (Lange calipers, Beta Technology Inc). Weight and height will be measured in duplicate to the nearest 0.1g and 0.1 cm, respectively, and averaged. All circumference and skinfold measurements will be taken in duplicate to the nearest 0.1mm; if the two measurements differ by more than 2mm, then we will collect a third measurement. All members of the research team will receive training to ensure measurements are standardized and accurate. Additionally, the initials of the research assistant taking the measurements will be noted on all anthropometric recording sheets to check for consistency between researchers. The infant measurements will be used to calculate infant weight-for-length, weight-for-age, length-for-age, BMI-for-age, head circumference-for-age, and mid-upper arm circumference-for-age z-scores based on the WHO standards [36]. To estimate infant fat mass using the skinfold measurements, we will utilize anthropometric equations created by Deierlein and colleagues [37] and Sen and colleagues [38] (Fig 2).

*Baby Eating Behavior Questionnaire (BEBQ)*. We will evaluate maternal perception of infant appetite, hunger, and satiety during breastfeeding using the Baby Eating Behavior Questionnaire (BEBQ) completed at 1, 3 and 4 months. The BEBQ consists of 18 questions ranked on a 5-point Likert scale: Never, Rarely, Sometimes, Often, or Always. The questionnaire has been used in previous research in Samoa by the OLaGA research team, validated for the population [39], and has been previously translated into Samoan. See Table 2 for more information on questionnaire and survey contents.

#### Home visit variables and procedures (Day 2)

*Breastfeeding diary*. Mothers will be asked to complete a 24-hour breastfeeding diary prior to each home visit. This will record the number of breastfeeding bouts and their length.

*Observation*. Breastfeeding observation will be used to measure milk intake over a full feed. Mothers will be asked to complete a full feed of the infant during the home visit. The home visit will take place in the morning (8am–12pm) to mirror the lab visit timings as closely as possible. Infant weight will be recorded twice at each visit: first prior to feeding and second after completion of full feed; these will be subtracted from each other as a proxy for human milk volume intake over the course of the feed.

*Infant feeding behavior and practices questionnaire*. A brief survey will collect information on infant feeding practices, such as average frequency and duration of breastfeeding bouts per day. The questions used on the survey have been adapted from the Infant Feeding Practices Study II [40]. Additionally, the survey will collect information on infant sleeping location and night time feeding schedule to record any co-sleeping and/or breast-sleeping practices. A silhouette instrument will be used to compare maternal perception of current infant body size versus ideal body size [41].

Data collected from the survey will be cross-checked with the 24-hour diary to compare answers regarding average frequency and duration. If large discrepancies are noted or feeding patterns arise that are outside of the biological norm, the participant will receive a telephone follow up call within 48 hours of the visit to be probed further to clarify. This step is due to concerns raised during pilot data analysis in which participants stated feeding patterns during survey collection that raised suspicion—for example, only feeding an exclusively breastfed infant twice daily.

The final portion of the questionnaire will explore maternal knowledge of infant hunger and feeding cues using open-ended questions to encourage participants to express their opinion on the topics presented. These answers will be voice-recorded to allow for later transcription, translation to English (if needed), and qualitative analysis. The initials of the research assistant will be noted at the beginning of all transcripts to check for potential researcher bias or leading questions in later analysis.

The questionnaire will be completed simultaneously alongside the breastfeeding observation. See Table 2 for more information on questionnaire and survey contents.

### Data management and protection of human subjects

Only the consent form will contain identifying information (name, contact number, and address), stored separately in a locked filing cabinet from all other data. All other data will be linked via a non-identifying ID number. Data records will be identified only through this unique ID number, no personally identifying information will be marked on any data or sample and will be stored in password-encrypted files only. Physical consent forms will be stored in a locked file cabinet at the OLaGA research center in Samoa during the data collection period and will be later transported and stored in a locked file cabinet at Yale University upon return. Questionnaire responses will be collected and managed using Research Electronic Data Capture (REDCap) tools hosted at Yale University [43, 44]. REDCap data will be entered using only the non-identifying participant ID and converted in an Excel file upon completion of all data collection. Upon return to the USA, data files will be uploaded to a purposefully created repository on Yale University’s secure servers.

Ethical considerations will be taken into account before beginning any research. As our research investigates the impact of maternal body composition on human milk hormonal concentration, care must be taken to ensure that our findings are not used to discourage women from breastfeeding or stigmatize body size in Samoa. Regardless of our findings, breastfeeding has many advantages to the mother and infant dyad that should be promoted, protected, and supported. Additionally, as the setting of the OLaGA facilities is within the MOH, participants may be led to believe that we are a medical facility that is able to offer healthcare assistance. Care will be taken to emphasize that we are not a medical facility and research outcomes will not provide a diagnosis nor should be treated as such. We will ensure that participants are reminded of the academic nature of the research and will refer the participant to a medical doctor or provide information on sources of support for breastfeeding if concerns are raised. Lastly, we will strive to ensure that monetary compensation for participation is an appropriate amount and does not elicit a coercive effect.

### Hormone analysis

Human milk and dried bloodspot samples will be shipped frozen (on dry ice) from Samoa to Yale University for hormonal analysis. Human milk samples will be defrosted at room temperature and milk fat separated from the aqueous phase by centrifugation at 2000x g for 20 minutes. The aliquots will then be skimmed by siphoning under the layer of fat. If needed, centrifugation and skimming will be repeated to ensure complete skimming. Dried bloodspot samples will be punched into 2x 3mm discs and reconstituted in assay buffer overnight at 4°^C^, and then placed on a plate shaker set at 350rpm for one hour.

All samples will be assayed using commercially available enzyme-linked immunosorbent assay (ELISA) kits for leptin [Leptin Ultrasensitive, ALPCO USA, Salem, NH] and adiponectin [Human Adiponectin, High Sensitivity, BioVendor R&D, Asheville, NC], and radioimmunoassay (RIA) kits for ghrelin [Human Ghrelin (Total), EMD Millipore, Darmstadt, Germany] following assay kit instructions.

### Statistical analysis

All exploratory data analyses and statistical analysis will be performed using R [45] or SPSS [46] software packages. Descriptive statistics will be produced for all results and specific analysis conducted to address each hypothesis. Data will be cleaned, with care taken to identify out of range or missing values and outliers. Variables, in particular metabolic hormone concentration results, that do not meet the criteria for parametric statistical assessment will be log-transformed before analyses. If found to still not follow the requirements for normal distribution or equal variances, non-parametric methods will be utilized. Univariate analysis (t-test, Pearson’s correlation) will be used first to test for differences in metabolic hormone levels or BEBQ scale outcomes with hypothesis specific variables. Secondly, multivariate analysis (multivariable linear regression, generalized linear models, repeated measures ANOVA, mixed-effects models with random intercepts) will test for differences incorporating multiple variables, controlling for covariates.

### Power considerations

Power calculations were calculated in G*Power 3.1 [47] using the limits α = 0.05, and β = 0.2 based on previous studies on maternal weight [6] and infant growth [48]. Based on these power calculations, a sample size of 80 mother-infant dyads was deemed feasible for addressing repeated measures differences in metabolic hormonal levels in human milk dependent on mother’s weight status or infant growth and body composition development.

More specifically, using data from Weyermann and colleagues [6], we determined that a sample size of 33 for each maternal BMI category (overweight and obesity) is required to detect differences in leptin and adiponectin levels with maternal weight status using a one-tailed repeated measures ANOVA. To account for possible loss to follow-up or cessation of breastfeeding during the data collection period, a sample size of 80 was chosen. The power analysis conducted for the second aim, using data from Fields and Demerath [45], determined that a sample of 50 infants is required to detect an association between infant growth and human milk leptin concentration using multivariate linear regression models.

While loss to follow-up is a known limitation of prospective cohort study design, we will endeavor to safeguard statistical power in our study through sufficient sample recruitment and brief intervals between sample collection points. Additionally, participants will receive spacing of the compensation for their time across each home visit (months 1, 3, and 4), as well as a small gift for the infant at the conclusion of the entire study period.

### Study timeline

Participant recruitment is predicted to begin in Spring 2023. Data collection will proceed on a rolling basis as qualified participants are identified. Data collection is estimated to last for one year (Spring 2023 –Spring 2024). Upon completion of data collection, all biospecimens will be shipped to Yale University, USA for analysis. Dissemination of results to participants will be coordinated with the designated MOH official by January 2025.

## Discussion

### Strengths and significance

This research will advance our understanding of human variation in the developmental programming of satiety and implications for early infant growth and body composition. While much is already known regarding the relationship between maternal BMI, human milk hormone concentration, and infant satiety, this study will be among the first research to characterize its relationship in women with higher BMI and to include a more comprehensive measurement of body composition through use of DXA scans. The use of a prospective longitudinal cohort alongside data collection that utilizes a mixed methods approach will allow us to capture a more accurate representation on both biological and cultural variables at play in the Samoan population, a historically underserved community.

We will work closely with the MOH Nutrition Surveillance Unit in Samoa, using the results of the research—alongside the pilot data collected in 2020–2021—to help create public health messages that aim to tailor culturally specific nutritional information about breast milk to mothers. These public health messages will aim to further promote breastfeeding in this population and attempt to counter the cycle of childhood obesity. Issues that may arise regarding concerns or worry over milk content following our research are addressed both previously in the methods and additionally in limitations below.

### Limitations

A limitation of our study is the finite collections of bloodspots samples that can be taken, the baselines of the mother’s circulating hormones and the infant’s circulating hormones are not a direct capture of the transfer happening through the milk at that moment. More frequent bloodspot collections would capture a more accurate representation of metabolic hormone transfer between mother and infant, however, multiple bloodspot collections from an infant are not feasible. This will be taken into consideration when evaluating the bloodspot data and drawing conclusion regarding the timing of hormonal transfer between mother and infant.

Similarly, we will only be collecting one human milk sample per time point. Human milk is a dynamic and multi-faceted fluid, and as such, varies both across the day (primarily due to circadian rhythms [49, 50]) and from day to day (due to variables such as external temperature, infant temperament, infection and illness, and many more [51]). While differences between days are outside of the scope of this study, we will control for timing of day through the restriction of human milk sample collection to between the hours of 8 and 10am.

Self-selection bias—such as higher educational status or household income—may be present because of our recruitment strategy, presenting a disproportionate representation of demographic backgrounds in our sample. To evaluate the degree of potential bias, we will collect data on sociodemographic characteristics such as educational level and employment status of the participants to compare with the same characteristics of the overall population.

### Local dissemination plans

We will give individual feedback to the mothers and infants using a feedback sheet at each data collection point (1, 3, and 4 months postpartum). This sheet will contain feedback on the infant’s weight and length status with an attached growth percentile chart. In terms of the overall study findings, we will first share the findings of this research with the Samoan MOH. This data dissemination and sharing will occur in three stages: (1) The research team will share all questionnaires and all de-identified, raw data with the designated MOH contacts; (2) Within a year of completing data collection, the research team will provide the MOH contact with cleaned data, a data cleaning log, and reports of the study findings; (3) Guided by the MOH we plan to use local media sources (newspaper, television) to disseminate key messages from the study findings to the wider community.

## Abbreviations

BEBQ: Baby Eating Behavior Questionnaire
BIA: Bioelectrical Impedance Analysis
BMI: Body Mass Index
DXA: Dual-energy X-ray Absorptiometry
ELISA: Enzyme-Linked Immunosorbent Assay
Hb1Ac: Glycosylated Hemoglobin A1c
MOH: Ministry of Health (Samoa)
NHS: National Health Services of Samoa
OLaGA: Obesity, Lifestyle, and Genetic Adaptations Laboratory (Apia, Samoa)
REDCap: Research Electronic Data Capture
RIA: Radioimmunoassay
